# The Class III Peroxidase (POD) Gene Family in Cassava: Identification, Phylogeny, Duplication, and Expression

**DOI:** 10.3390/ijms20112730

**Published:** 2019-06-03

**Authors:** Chunlai Wu, Xupo Ding, Zehong Ding, Weiwei Tie, Yan Yan, Yu Wang, Hai Yang, Wei Hu

**Affiliations:** 1 Key Laboratory of Biology and Genetic Resources of Tropical Crops of Ministry of Agriculture, Institute of Tropical Bioscience and Biotechnology, Chinese Academy of Tropical Agricultural Sciences, Haikou 571101, China; wuchunlai19900109@126.com (C.W.); dingxupo@itbb.org.cn (X.D.); dingzehong@itbb.org.cn (Z.D.); tieweiwei@itbb.org.cn (W.T.); yanyan@itbb.org.cn (Y.Y.); 2 The Genetic Engineering International Cooperation Base of Chinese Ministry of Science and Technology, College of Life Science and Technology, Huazhong University of Science and Technology, Wuhan 430074, China; 3 Beijing Commerce and Trade School, Beijing 100162, China; wangyu7071@126.com; 4 National Engineering Research Center for Nanomedicine, College of Life Science and Technology, Huazhong University of Science and Technology, Wuhan 430074, China

**Keywords:** expression, genome-wide, identification of peroxidase genes, duplication pattern, stress, cassava

## Abstract

The class III peroxidase (POD) enzymes participate in plant development, hormone signaling, and stress responses. However, little is known about the POD family in cassava. Here, we identified 91 cassava *POD* genes (*MePODs*) and classified them into six subgroups using phylogenetic analysis. Conserved motif analysis demonstrated that all MePOD proteins have typical peroxidase domains, and gene structure analysis showed that *MePOD* genes have between one and nine exons. Duplication pattern analysis suggests that tandem duplication has played a role in *MePOD* gene expansion. Comprehensive transcriptomic analysis revealed that *MePOD* genes in cassava are involved in the drought response and postharvest physiological deterioration. Several *MePODs* underwent transcriptional changes after various stresses and related signaling treatments were applied. In sum, we characterized the POD family in cassava and uncovered the transcriptional control of *POD* genes in response to various stresses and postharvest physiological deterioration conditions. These results can be used to identify potential target genes for improving the stress tolerance of cassava crops.

## 1. Introduction

Peroxidases (EC 1.11.1.X) form a large family of enzymes that are widely distributed in living organisms and catalyze the oxidoreduction reaction between hydrogen peroxide (H_2_O_2_) as an electron acceptor and diverse electron donors, such as auxin, phenolic compounds, or secondary metabolites [1,2]. According to their protein sequences and structure, peroxidases are classified as either non-heme peroxidases or heme peroxidases [3]. The majority of heme peroxidase members are divided into animal and non-animal groups [4]. On the basis of their amino acid sequences and catalytic properties, non-animal heme peroxidases are assigned to one of three large families: class I, II, or III [3,5]. The class III peroxidases (EC 1.11.1.7) are plant-specific oxidoreductases and have various abbreviations (POX, POD, Px, PER, and Prx) [2].

There are many multigenic class III peroxidasesin land plants, which is commonly secreted into the vacuole and cell wall [4,5,6,7]. The structures and weights of Prx proteins are highly conserved between paralogs and orthologs [1,3]. The class III plant peroxidases contain 10–12 conserved α-helices and two short β-strands [2,7,8,9,10], and they mainly participate in the peroxidative cycle and hydroxylic cycle to reduce the production of hydrogen peroxide and the formation of reactive oxygen species (ROS) [4,5,11,12]. Prx proteins are involved in a variety of physiological processes, such as the cross-linking of cell wall components, salt tolerance, defense against pathogen attack, the oxidation of toxic reductants, and the metabolism of phytohormones [2,3,13,14,15,16,17].

Some genetic evidence supports Prx proteins’ role in the plant response to biotic and abiotic stresses. Overexpression of *AtPrx64* was able to enhance tolerance to aluminum stress in transgenic tobacco plants [18]. *AtPrx3* was shown to positively regulate plant tolerance to drought and salt stresses in Arabidopsis [19]. Overexpression of the *Catharanthus roseus* genes *CrPrx* and *CrPrx1* in tobacco led to enhanced chilling resistance and increased germination rates under dehydration and salt treatments, respectively [20]. Repressing the expression of *Ep5C* in tomato resulted in reduced susceptibility to bacterial speck caused by the pathogen *Pseudomonas syringae* pv *tomato* [21]. *CaPO2* gene-silenced pepper plants were shown to be susceptible to infection by *Xanthomonas campestris* pv *vesicatoria*, whereas overexpression of *CaPO2* in transgenic *Arabidopsis thaliana* conferred bacterial disease resistance [22]. Transgenic carrot plants overexpressing *OsPrx114* exhibited enhanced resistance to necrotrophic fungal pathogens [23]. Together, these previous studies reveal the positive role of class III plant peroxidases in the response to biotic and abiotic stresses.

To date, the peroxidase (POD) family members have been characterized by whole-genome analyses in several plants, including 73 PODs in Arabidopsis [24,25,26], 138 PODs in rice [9], 93 PODs in *Populus trichocarpa* [27], 102 PODs in *Medicago sativa* [28], 119 PODs in maize [29], and 94 PODs in *Pyrus bretschneideri* [30]. However, there is less known about the POD family in cassava, a major tropical crop. Cassava is the third most valuable crop after maize and rice in Africa, Latin America, and Asia, supplying a carbohydrate source to 600 million people in tropical and subtropical regions [31]. Cassava can efficiently use water, heat, and light resources, and it is resistant to dehydration stress and lower-fertility soils [32,33]. Unfortunately, the potential of cassava as a food and industrial crop is restricted because its storage roots deteriorate within 72 h of its harvest [34]. ROS production is an early event that leads to the postharvest physiological deterioration (PPD) of cassava storage roots [35]. The mechanisms underlying cassava’s resistance to drought and sensitivity to PPD are not well understood. POD proteins function by reducing the production of H_2_O_2_ and formation of ROS, which are involved in various physiological processes. Systematic investigations of the cassava POD family would provide novel insights into the POD-mediated stress response and regulation of root deterioration.

## 2. Results

### 2.1. Genome-Wide Identification of PODs in Cassava

According to the 211 POD protein sequences from Arabidopsis and rice genome databases, 91 POD members were predicted from the cassava genome using BLAST and HMMER methods. After conserved domain detection was confirmed, these cassava POD proteins (MePODs) were named MePOD01 to MePOD91. The full length of these putative cassava POD proteins ranges from 153 (MePOD63) to 422 (MePOD46) amino acid residues, and their relative molecular weight varies from 16.64 (MePOD63) to 46.12 kDa (MePOD46), with isoelectric points ranging from 4.43 (MePOD26) to 9.63 (MePOD39) (Table S1).

### 2.2. Phylogenetic and Comparative Analyses of PODs in Cassava

**Figure 1:**
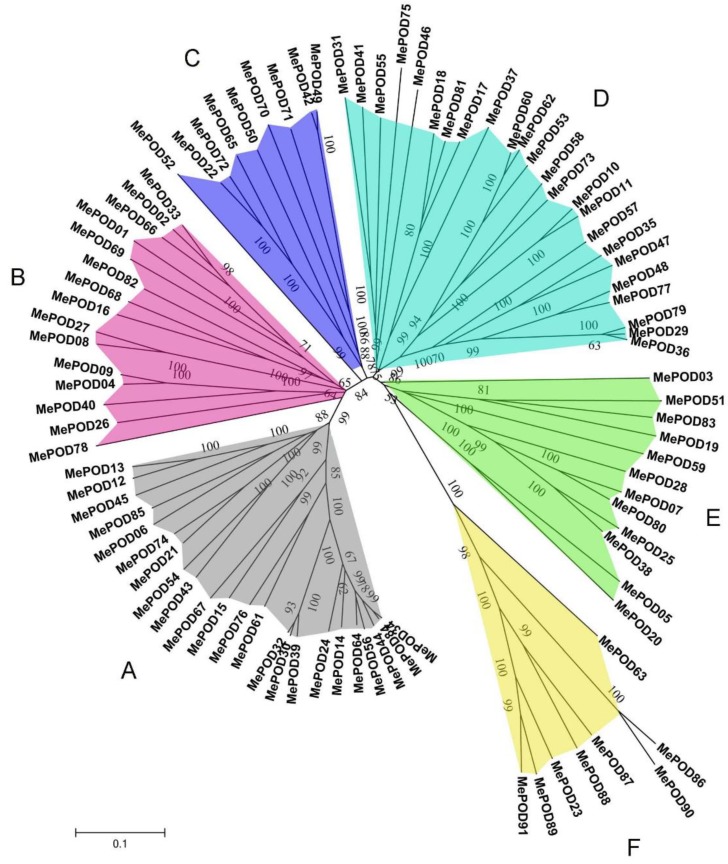
Phylogenetic analyses of PODs from cassava. A total of 91 PODs from cassava were used to create the neighbor-joining (NJ) tree with 1000 bootstraps.

The homology and similarity of the *POD* genes in cassava were determined by performing multiple sequence alignments. Then, the radiation phylogenetic tree of the 91 *POD* genes was constructed using the neighbor-joining (NJ) method with a bootstrap value of 1000 using MEGA 5.1 (University College Dublin, Dublin, Ireland). The phylogenetic analyses indicated that *MePOD* genes can be divided into six subgroups on the basis of the observed genetic distance and bootstrap support (Figure 1). The large subgroups A and D consist of 23 and 24 MePOD members, respectively, whereas the small subgroups C and F contain 9 and 8 MePOD members, respectively. Subgroups B and E are composed of 15 and 12 MePODs members, respectively. These results show that a diversified POD family exists in cassava.

### 2.3. Conserved Motif and Gene Structure Analysis of POD Families in Cassava

**Figure 2:**
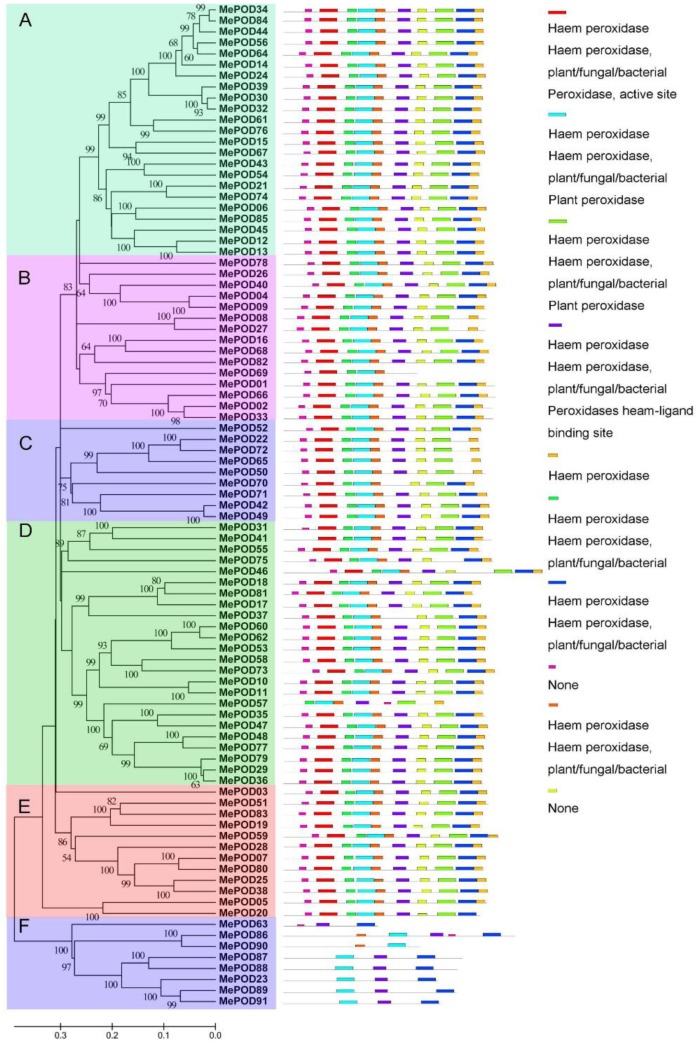
The motif analyses of POD family members in cassava according to their evolutionary relationship. The POD motifs were identified by the MEME database. The 10 different colors of the boxes on the right represent diverse conserved motifs, while the gray lines indicate non-conserved sequences.

The structural features of MePODs were investigated by identifying 10 conserved motifs using the MEME database in accordance with the phylogenetic relationship. Then, the conserved motifs were submitted in their entirety to the InterProScan database for annotation. Eight domains (domains 1, 2, 3, 4, 5, 6, 7, and 9) were noted as POD protein motifs, which are an essential feature of the peroxidase family. On the basis of the motif analyses, 83 MePODs were assigned to one of five subgroups (A–E). Each of these 83 MePODs contains at least nine POD motifs, except for MePOD57 (in subgroup D) and MePOD69 (in subgroup B), which have seven and five motifs, respectively. The presence of these motifs suggests that the identified proteins are characteristic of the POD family (Figure 2). Subgroup F is distinct from the others: its members contain domains 2, 4, 5, 7, and 8. These results indicate that the proteins assigned to the same subfamilies share similar POD motif characteristics, further supporting their phylogenetic classification as PODs in cassava.

**Figure 3:**
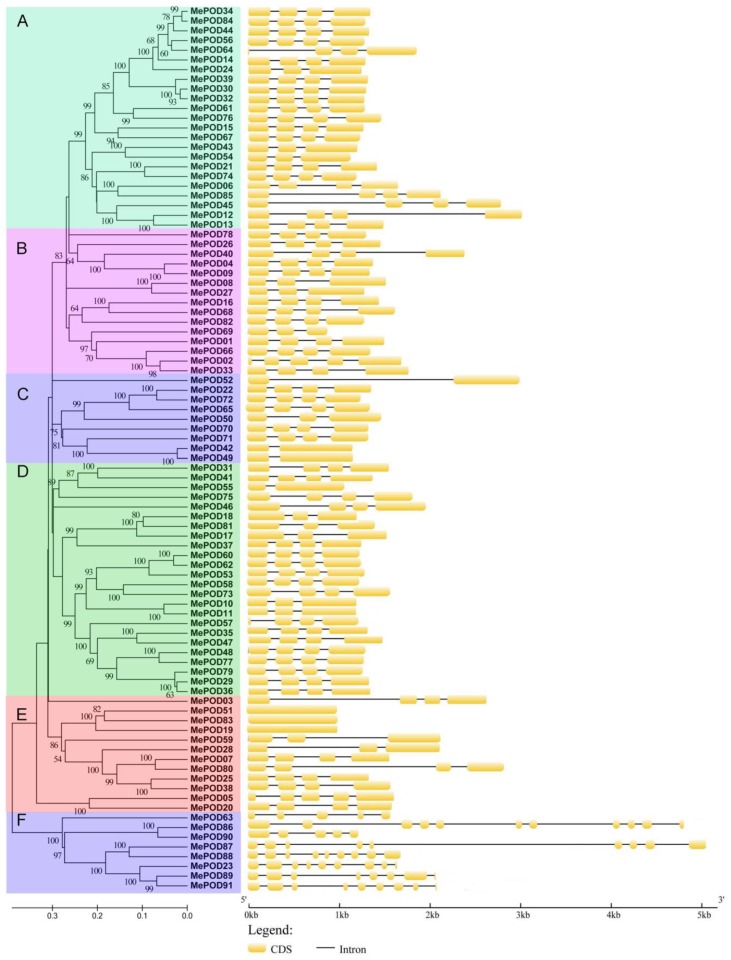
The exon–intron organization analyses of cassava PODs on the basis of the phylogenetic relationship. The exon–intron distribution was established using the GSDS database. The yellow boxes and the black lines represent exons and introns, respectively.

Next, the exon–intron structures of cassava *POD* genes were analyzed. Subgroup F is exon-rich, with five to nine exons, whereas other subfamilies have fewer (between one and four) exons, except for *MePOD02* (in subgroup B), which has five exons (Figure 3). High proportions of *POD* genes contain four exons in subgroups A, B, C, and D, with four-exon *POD* genes forming 84%, 67%, 57%, and 76% of the genes in these subgroups, respectively, whereas only 50% of the subgroup E genes have four exons. Generally, *POD* genes in the same subgroup show similar exon–intron features, providing further evidence of their phylogenetic relationship.

### 2.4. Analyses of Chromosomal Distribution and Duplication Events of the Cassava *POD* Genes

**Figure 4:**
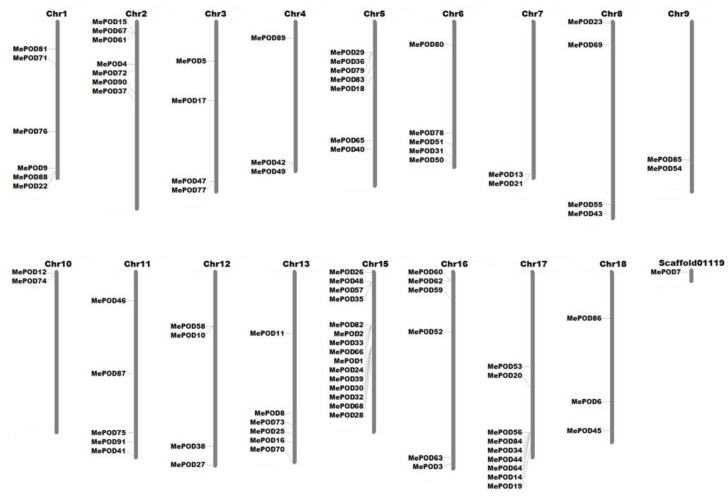
Chromosome distribution analyses of *POD* gene subgroups in cassava. The chromosomal information of 91 MePODs was collected from the Phytozome 12.0 cassava database, and the genes were then mapped to 17 chromosomes and one scaffold. MapInspect software (Wageningen University, Wageningen, Netherlands) was used to draw this figure.

The locations of the cassava *POD* genes were determined by analyzing their chromosomal distribution (Figure 4). The 91 MePODs were mapped to chr1, 2, 3, 4, 5, 6, 7, 8, 9, 10, 11, 12, 13, 15, 16, 17, and 18, and scaffold01119. The 24 *POD* genes in subgroup D were distributed among chr1, 2, 3, 5, 6, 8, 11, 12, 13, 15, 16, and 17, making subgroup D the most widely distributed subgroup. Subgroup F contains eight *POD* genes, which are located on chr1, 2, 4, 8, 11, 16, and 18; thus, of the six subgroups identified, subgroup F is dispersed among the fewest chromosomes. The 23 members of subgroup A are found on chr1, 2, 7, 8, 9, 10, 15, 17, and 18, among which chromosomes 7, 9, and 10 only contain subgroup A genes. Generally, the cassava *POD* genes are widely distributed among chromosomes.

**Figure 5:**
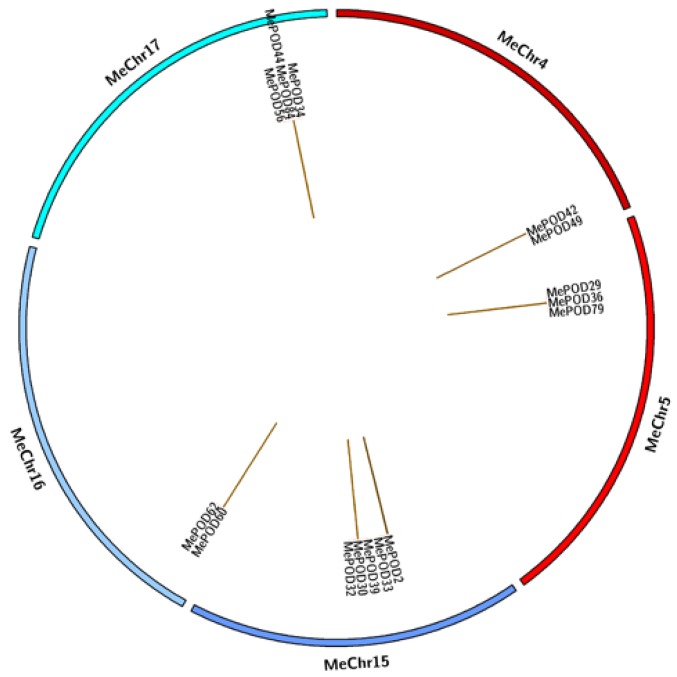
Analyses of *POD* gene duplication in cassava. The program Circos (Canada’s Michael Smith Genome Sciences Center, Vancouver, Canada) was used to draw different chromosomes in a circular distribution. The brown connection lines represent tandem duplication events of *POD* genes in cassava.

To further investigate the expansion of *POD* genes in cassava, we aligned the total nucleotide sequences of the 91 *MePOD* genes to identify duplication events. We identified 15 events involving 16 paralogs (*MePOD2/MePOD33*, *MePOD29/36/79*, *MePOD30/32/39*, *MePOD34/44/56/84*, *MePOD42/MePOD49*, *MePOD60/MePOD62*), suggesting that tandem duplication played a significant role in POD family expansion in the cassava genome (Figure 5).

Next, we calculated nonsynonymous (Ka) and synonymous (Ks) ratios to understand the modes of evolutionary selection for the duplicated *MePOD* genes. We found that the Ka/Ks ratios of the paralogous genes are between 0.05 and 0.29, indicating that these genes underwent purifying selection during evolution (Table S2).

### 2.5. Expression Profiles of POD Genes in Different Tissues of Two Cassava Genotypes

**Figure 6:**
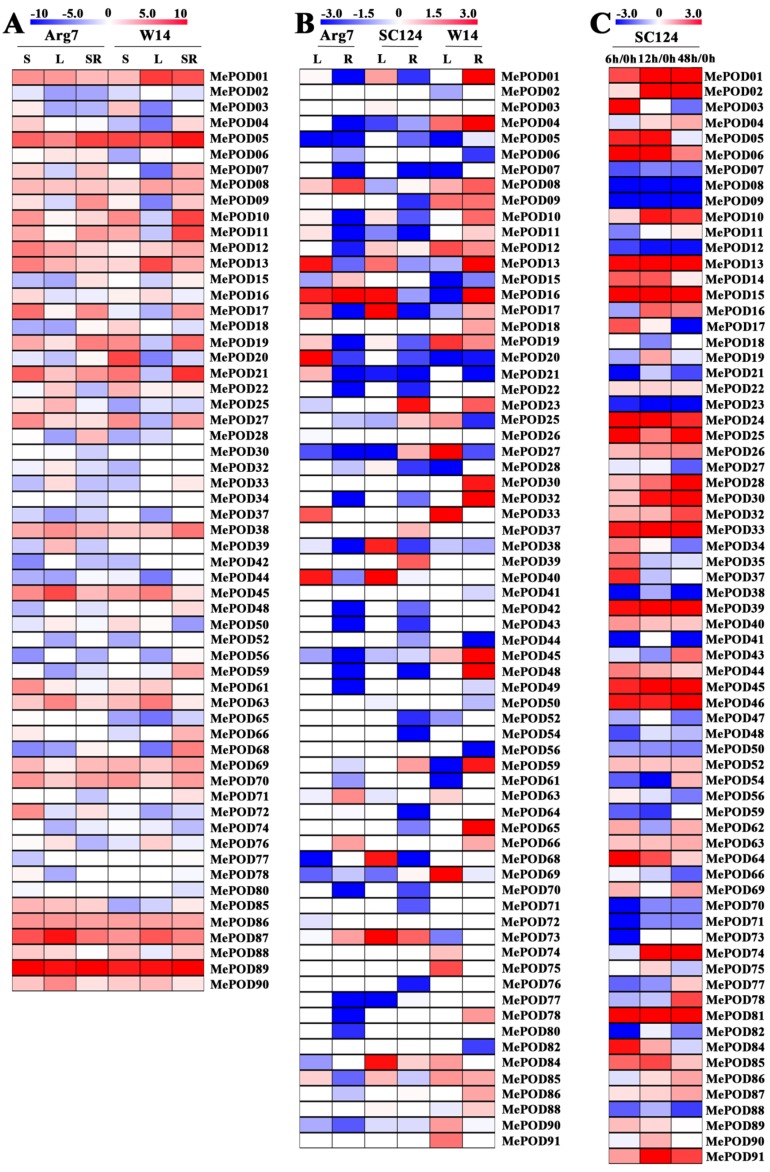
Transcriptomic analysis of cassava *POD* genes. (**A**) Expression of MePODs in the stem (S), leaf (L), and storage root (SR) of W14 and Arg7. The log2-based FPKM value was applied to build the heat map using Mev4.9.0 software (CCCB, Boston, USA). (**B**) Expression of MePODs in the leaf (L) and root (R) of Arg7, SC124, and W14 after drought treatment relative to under normal conditions. Log2-based fold changes (L/control; R/control) were applied to build the heat map using Mev4.9.0 software. (**C**) Expression of MePODs in the storage root at 6, 12, and 48 h relative to 0 h after harvest. Log2-based fold changes were applied to build the heat map using Mev4.9.0 software.

The expression levels of *MePOD* genes in different tissues were investigated by performing RNA-Seq analysis on the storage roots, stems, and leaves of a cultivated variety (Arg7) and wild subspecies (W14). The resulting expression data covered 59 and 56 *MePOD* genes in the transcriptome dataset of Arg7 and W14, respectively (Figure 6A; Table S3). Of these genes in Arg7, 15 (25%), 9 (15%), and 8 (14%) *MePODs* had high transcriptional levels (log2-based > 4) in stems, leaves, and storage roots, respectively. The number of *MePODs* with high expression (log2-based > 4) in the stems, leaves, and storage roots of W14 was 9 (16%), 7 (13%), and 10 (18%), respectively. Notably, *MePOD5* in subgroup E and *MePOD89* in subgroup F were strongly expressed (log2-based fold change > 4) in the three diverse tissues of Arg7 and W14. These *POD* genes may play a molecular role in the development and function of different cassava tissues.

### 2.6. Expression Profiles of POD Genes After Drought Treatment

To study the possible role of MePODs in the cassava response to drought stress, water was withheld from a wild subspecies (W14) and two cultivated varieties (Arg7 and SC124) for 12 days. The leaves and roots of these samples were then collected to perform RNA-Seq. Of the transcriptome data, the expression data were obtained for 71 out of the 91 cassava *POD* genes (Figure 6B; Table S4). After drought treatment, 6 (8%) and 5 (7%) *MePOD* genes in Arg7 were transcriptionally upregulated (log2-based fold change > 1), whereas 7 (10%) and 29 (41%) were down-regulated (log2 based fold change < −1) in the leaves and roots, respectively. After SC124 was subjected to drought stress, 9 (13%) and 4 (6%) *MePOD* genes were upregulated (log2-based fold change > 1) but 6 (8%) and 29 (41%) were downregulated (log2-based fold change <−1) in the leaves and roots, respectively. After the W14 subspecies was exposed to drought, 13 (18%) and 21 (30%) *MePOD* genes were induced (log2-based fold change > 1), whereas 11 (15%) and 9 (13%) were depressed (log2-based fold change < −1) in the leaves and roots, respectively. *MePOD13* (subgroup A) and *MePOD16* (subgroup B) were upregulated (log2-based fold change > 1) by drought stress in the leaves of all three genotypes. The above data reveal that more *MePOD* genes were upregulated in response to drought treatment in W14 than in Arg7 and SC124.

### 2.7. Expression Profiles of Cassava PODs During PPD

Transcriptome analyses were conducted at different postharvest periods of the storage roots of SC124 to examine the possible function of *MePOD* genes during PPD (Figure 6C; Table S5). Transcriptional data were obtained for 71 of the 91 MePODs. Of these MePODs, 38 genes were induced (log2-based fold change > 1) at a minimum of one time point. Notably, *MePOD-1, -6, -13, -15, -24, -25, -33, -39, -45, -46, -81,* and *-91* were continuously upregulated (log2-based fold change > 1) at all points. Conversely, *MePOD-7, -8, -9, -12, -23, -38, -50, -70, -71,* and *-88* were downregulated (log2-based fold change < −1) at all tested times. These results suggest the possibility that MePODs play a role during PPD in cassava.

### 2.8. Expression Analysis of MePOD Genes in Response to Various Abiotic and Biotic Stresses and Related Signals

**Figure 7:**
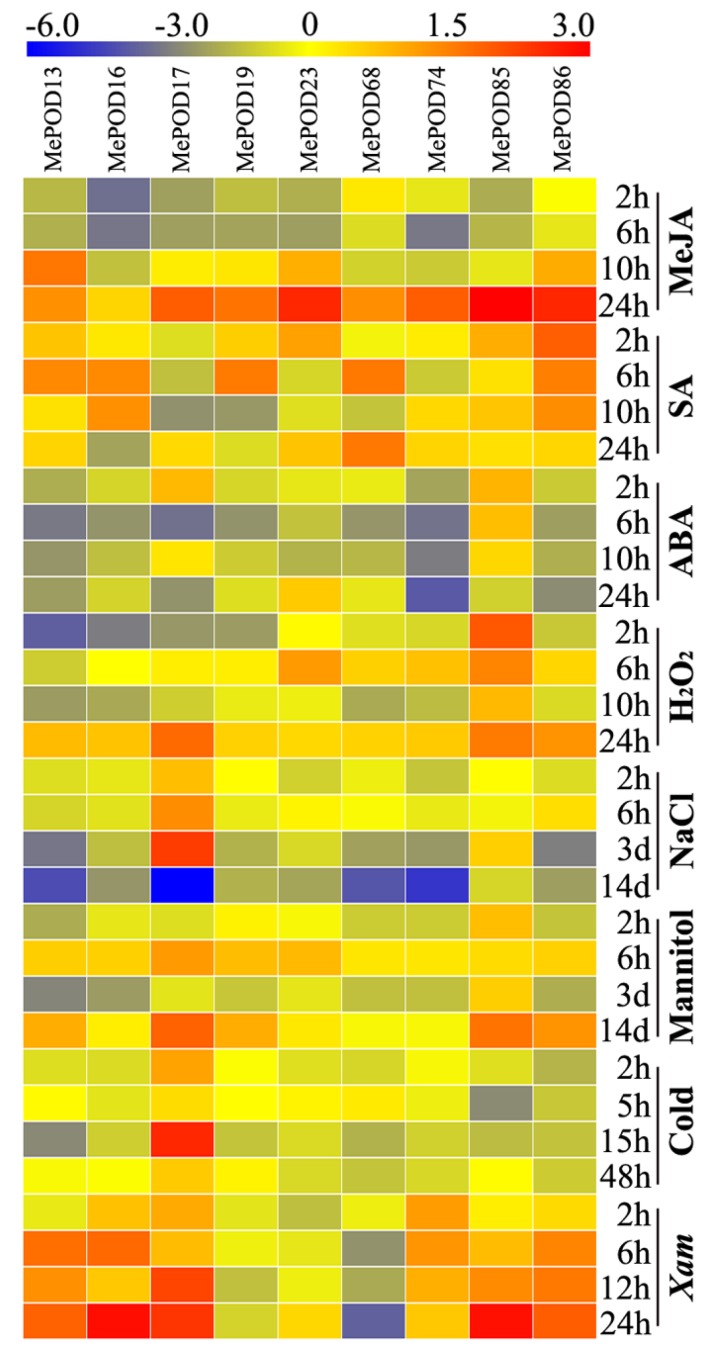
Expression profiles of cassava *POD* genes in the leaves of Arg7 after exposure to MeJA, SA, ABA, H_2_O_2_, salt, osmotic stress (mannitol treatment), cold stress, and *Xam*. Log2-based qRT-PCR fold changes were used to build the heat map with Mev4.9.0 software. The changes in color represent the relative gene expression level.

To test the transcription of *MePOD* genes upon exposure to methyl jasmonate (MeJA), salicylic acid (SA), abscisic acid (ABA), H_2_O_2_, salt, osmotic stress (by mannitol treatment), cold stress, and *Xanthomonas axonopodis* pv *manihotis* (*Xam*), nine genes (*MePOD-13, -16, -17, -19, -23, -68, -74, -85, -86*) that were induced by drought stress in at least two tissues in all three varieties were selected for quantitative real-time PCR (qRT-PCR) analysis (Figure 7; Table S6). We define upregulation as: log2-based fold change > 1. With MeJA treatment, the expression of eight genes (*MePOD-13, -17, -19, -23, -68, -74, -85,* and *-86)* was induced, with particularly high levels expressed 24 h after treatment. ABA treatment resulted in increased transcript levels of *MePOD-17* and *-85*. With SA treatment, the expression levels of *MePOD-13, -16, -19, -23, -68, -85,* and *-86* were amplified. H_2_O_2_ treatment led to the induction of *MePOD-13, -17, -23,* and *-85*. Salt treatment induced the expression of *MePOD17* after two and six hours, and three days of treatment, but the gene was repressed after 14 days of treatment. Under osmotic stress, *MePOD-13, -17, -19, -23, -85,* and *-86* were upregulated, among which *MePOD-85* was induced throughout the entire treatment time. In response to cold treatment, *MePOD17* was upregulated at 2 and 15 h. Exposure to the pathogen *Xam* led to the upregulated expression of six *POD* genes (*MePOD-13, -16, -17, -74, -85,* and *-86*) at a minimum of two time points. Together, these results demonstrate that the *POD* genes of cassava respond to multiple stresses and related signals (Figure 7).

## 3. Discussion

Given the significant role of PODs in various physiological processes, including responses to biotic and abiotic stresses, it was necessary to scientifically investigate the potential functions of *POD* genes in cassava, which is an important crop. In this study, we identified 91 PODs in the cassava genome (Figure 1); thus, cassava has more POD members than Arabidopsis but fewer than rice, *Populus trichocarpa, Medicago sativa*, maize, and *Pyrus bretschneideri* [9,24,25,26,27,28,29,30]. We found that 92% (84/91) of the MePODs have a molecular mass in the range of 30 to 45 kDa, which is in accordance with previous studies [2,7]. Most of the *POD* genes (89/91) in cassava harbor more than one exon (Figure 3), which is similar to the proportion of single-exon *POD* genes in *Pyrus bretschneideri* and *Zea mays* (*PbPRX* (90/94) and *ZmPRX* (89/107), respectively) [29,30]. The similarities in gene structure and motif composition among the members in each MePOD subgroup support the phylogenetic classification presented here.

The expansion of a gene family primarily occurs via three kinds of modes: segmental duplication of multiple genes, tandem duplication of individual genes, and whole-genome duplication [36,37]. To analyze the duplication modes of the *POD* genes in cassava, we first identified the chromosomal locations of the *MePOD* genes. Chromosomal mapping revealed that these genes are widely distributed among 17 chromosomes and one scaffold (cassava has 18 chromosomes in total) (Figure 4), which is in accordance with the wide chromosomal distribution of PODs in Arabidopsis, rice, *Populus trichocarpa*, maize, and *Pyrus bretschneideri* [9,24,27,29,30]. Secondly, 16 paralogous *POD* genes were characterized in the cassava genome, indicating that tandem duplication contributed to MePOD expansion. Accumulated evidence has demonstrated that duplication events have been important for gene expansion in the POD family. A total of 37 PRX genes in *Populus* and 24 *POD* genes in maize were identified as tandem duplications, further supporting that tandem duplication has been a significant means of *POD* gene expansion [27,29]. Almost all these paralogous MePODs had low or no expression after drought treatment, but 63% (10 out of 16) from the postharvest transcriptome were expressed (Table S6), of which *MePOD-2, -30, -32, -33, -39,* and *-44* were significantly upregulated and *MePOD-34, -56,* and *-62* were repressed at some time point during the PPD process (Figure 6C). These results indicate that most of the *MePOD* genes resulting from tandem duplication-driven expansion are involved in the PPD process of cassava storage roots. Paralogous PRX genes were also found to be involved in other biological processes. In maize, paralogous genes *ZmPRX-26, -42,* and *-75* were induced after NaCl, PEG, SA, or H_2_O_2_ treatment [29]. In Chinese pear, the expression of paralogous genes *PbPRX-42* and *-64* increased during fruit development [30].

The POD family is positively related to the reduced production of hydrogen peroxide and the decreased formation of reactive oxygen species, and the suppression of these species increases plant resistance to stresses [4,5,11,12,19,20]. In this study, the total number of *POD* genes responding to drought (log2-based fold change > 1) was greater in both the roots and leaves of W14 than that in Arg7 and SC124, suggesting the comprehensive activation of PODs in response to drought in W14 (Figure 6B). The wild ancestor W14 has been previously confirmed to be more resistant to drought than the two cultivars SC124 and Arg7 [38,39]. Accumulated evidence suggests that the overexpression of *POD* genes results in increased plant tolerance to drought and osmotic stresses [19,20,40,41]. The activity of POD enzyme was significantly enhanced under drought stress [42]. Consequently, we conclude that the high ratio of MePODs induced by drought in W14 might contribute to its strong drought tolerance.

Previous studies have suggested that ROS production results in the deterioration process in cassava during the postharvest period, and a reduction in ROS accumulation could delay the PPD process [35,43]. The POD family mainly participates in the peroxidative cycle and hydroxylic cycle, resulting in the reduced production of H_2_O_2_ and the decreased formation of ROS [4,5,11,12]. Some PRXs have been shown to change in expression during the fruit storage process [44,45]. The activity of POD enzyme significantly increased during cassava PPD process, suggesting their possible role during the postharvest period of cassava [35,44]. In this study, we found that 78% (71 out of 91) of PODs (log2-based fold change > 1) were upregulated in the storage roots of SC124 (Figure 6C). Interestingly, 13% (12 out of 91) of PODs (log2-based fold change > 1) were induced at all points. Collectively, these results indicate that *MePOD* genes are involved in the PPD process in cassava storage roots.

Previous research has indicated that PODs can extensively participate in plants’ responsse to biotic and abiotic stresses [18,19,20,21,22,23]. Here, we selected nine genes (*MePOD-13*, *-16*, *-17*, *-19*, *-23*, *-68*, *-74*, *-85*, and *-86*) to further examine their expression levels after various treatments (Figure 7; Table S6). These genes are located on different regions of chr7, 13, 3, 17, 8, 15, 10, 9, and 18, respectively (Figure 4). Phylogenetic analysis indicates that *MePOD-16*, *-68*, *-74*, and *-85* belong to subgroup A; *MePOD-17* belongs to subgroup D*;* and *MePOD-13*, *-19*, *-23*, and *-86* belong to subgroup E (Figure 1). The results show that all nine of the analyzed MePODs were upregulated in response to at least two types of treatments. *MePOD17* and *MePOD85* (log2-based fold change > 1) were induced by six treatments (MeJA, salt, cold stress, osmotic stress, ABA, and *Xam* and MeJA, osmotic stress, SA, ABA, H_2_O_2_, and *Xam*, respectively); *MePOD13* was upregulated by five treatments (MeJA, osmotic stress, ABA, SA, H_2_O_2_, and *Xam*); and *MePOD23* and *MePOD86* were upregulated by four treatments (MeJA, osmotic stress, SA, and H_2_O_2_ and MeJA, SA, H_2_O_2_, and *Xam*, respectively). Of these, *MePOD13* and *MePOD23* were induced after H_2_O_2_ treatment in cassava leaves (Figure 7) but exhibited the opposite trend of expression during the PPD process in storage roots (Figure 6C), suggesting their differential roles in diverse tissues. *MePOD-13, -19, -23, -68,* and *-86* (belonging to subgroup E, except for *MePOD68*) were upregulated by MeJA and SA treatments but downregulated by ABA treatment (Figure 1 and Figure 7). The expression of some PODs has been induced by MeJA and SA treatments in other plant species [2,46]. The opposite direction of expression of these *POD* genes between MeJA and SA treatments and ABA treatment may be due to the antagonism between MeJA/SA and ABA [47,48]. Whereas ABA plays a prominent role in plants’ tolerance to drought stress [38], MeJA- and SA-mediated signaling pathways are also activated under drought stress [49,50]. The induction of these genes by MeJA, SA, and drought suggests their possible involvement in MeJA- and SA-mediated drought responses in cassava. The responses of *POD* genes to multiple treatments have been observed in other plants. In Arabidopsis, *AtPrx33* and *AtPrx34* were upregulated after H_2_O_2_ and flg22 treatments [51]. In maize, *ZmPRX-26*, *-42*, and *-71* were induced by H_2_O_2_, salt, and PEG treatments [29]. Phylogenetic analysis of MePODs with AtPrx-33 and -34 and ZmPRX-26, -42, and -71 found that MePOD86 shares a close phylogenetic relationship with ZmPRX71 (Figure S1), suggesting their functional conservation in multiple treatments. Multiple stresses, such as cold, salt, or PEG, induced the activity of POD enzyme, demonstrating the response of *POD* genes to environmental stress [52,53,54]. These results suggest that MePODs participate in the response to multiple stresses or related signals and are candidate targets for the genetic improvement of cassava.

## 4. Materials and Methods

### 4.1. Plant Materials and Treatments

Three cassava genotypes, W14, SC124, and Arg7, were planted in the greenhouse of the Chinese Academy of Tropical Agricultural Science (Haikou, China). Their characteristics were described in our previous studies [38,55]. Stem segments containing three nodes were cut from eight-month-old cassava plants and planted in pots, as described in Hu’s study [51]. The transcripts of W14 and Arg7 *MePOD* genes in stems and leaves after being planted for 90 days and middle storage roots after being planted for 150 days were examined by RNA-Seq. After W14, SC124, and Arg7 were cultured for 90 days, they were subjected to drought stress by withholding water for 12 days, after which their leaves and roots were sampled to study the transcriptional responses by RNA-Seq. To examine the expression profiles of *MePOD* genes after the plants were exposed to stress and related signaling treatments, the 60-day-old Arg7 variety was treated with 100 μM MeJA for 0, 2, 6, 10, and 24 h; 300 mM NaCl for 0 h, 2 h, 6 h, 3 days, and 14 days; a low temperature (4 °C) for 0, 2, 5, 15, and 48 h; 100 μM SA for 0, 2, 6, 10, and 24 h; 200 mM mannitol (to induce osmotic stress) for 0 h, 2 h, 6 h, 3 days, and 14 days; 100 μM ABA for 0, 2, 6, 10, and 24 h; 10% H_2_O_2_ for 0, 2, 6, 10, and 24 h; or *Xam* for 0, 2, 6, 12, and 24 h. Ten-month-old cassava storage roots (CSR) were cut into 5-mm-thick slices and placed into Petri dishes containing wet filter paper for 0, 6, 12, and 48 h to study the expression changes in *MePOD* genes via RNA-Seq during CSR deterioration. All samples were frozen immediately in liquid nitrogen and stored at −80 °C for RNA-Seq and qRT-PCR.

### 4.2. Identification and Phylogenetic Analysis of PODs in Cassava

*MePOD* genes were identified in cassava on the basis of homology with 73 POD protein sequences from the *Arabidopsis* genome database (available online: http://www.arabidopsis.org/index.jsp) and 138 POD protein sequences from the rice genome database (available online: http://rice.plantbiology.msu.edu/index.shtml) [56,57]. The Hidden Markov Model-based search (HMMER: http://hmmer.wustl.edu/) profile of these confirmed POD proteins was constructed to search the cassava genome hub (available online: http://www.phytozome.net/cassava.php) [58]. Finally, all predicted POD protein sequences were further examined by CDD (available online: http://www.ncbi.nlm.nih.gov/cdd/) and PFAM (available online: http://pfam.sanger.ac.uk/) after being checked by BLAST analyses [59,60]. All the predicted cassava POD genes identified from HMMER and BLAST were confirmed only if they included the POD special domains examined by SMART (available online: http://smart.embl-heidelberg.de/) [61]. Multiple sequence alignment of all predicted MePOD protein sequences was performed with Clustal W in BioEdit software [62]. The phylogenetic tree of the full-length MePOD protein sequences was created using MEGA 5.0 (University College Dublin, Dublin, Ireland) with the neighbor-joining (NJ) method, and bootstrap analysis was conducted with 1000 replicates [63].

### 4.3. Protein Properties and Structure Analyses of PODs in Cassava

The ProtParam database (available online: http://web.expasy.org/protparam/) was used to predict the properties, including amino acid numbers, molecular weights (MW), and isoelectric points (pI), of all presumed POD proteins [64]. The motifs were analyzed using the MEME program (available online: http://meme-suite.org/tools/meme), in which the maximum number of motifs was set to 10, the optimum width of motifs was set to 15–50 amino acid residues, and the other settings were kept at default values [65]. Subsequently, these 10 motifs were annotated in InterProScan (available online: http://www.ebi.ac.uk/Tools/pfa/iprscan/) [66]. The gene structures of each MePOD were investigated using GSDS software (available online: http://gsds.cbi.pku.edu.cn/) using each MePOD’s genomic DNA sequence and its corresponding CDS sequence, which were retrieved from the cassava genome database [67].

### 4.4. Chromosomal Location and Duplication Pattern Analyses

According to the results of BLASTN in the Phytozome 12.0 cassava database, *MePOD* genes were mapped to different chromosomes. On the basis of the calculated value of nucleotide sequence similarity and the phylogenetic relationship of cassava *POD* genes, paralogous genes were identified. The gene duplication pattern of paralogous *MePOD* genes was determined by the following two criteria: (1) the identity of the aligned region was >90% and (2) the alignment covered >90% of the longer gene. Circos software (Canada’s Michael Smith Genome Sciences Center, Vancouver, Canada) was used to draw the duplication events of *MePOD* genes [68,69]. The values of nonsynonymous substitution (Ka) and synonymous substitution (Ks) were calculated suing DnaSP 5.0 software [70]. Ka/Ks rate > 1 indicates positive evolution, Ka/Ks rate = 1 indicates neutral evolution, and Ka/Ks rate < 1 indicates negative evolution [71].

### 4.5. Transcriptome Analyses of PODs in Cassava

RNA-Seq was used to determine the expression of cassava *MePOD* genes. Total RNA was isolated from frozen stems, leaves, roots, and storage roots using the plant RNeasy extraction kit (TIANGEN, Beijing, China) and quantified with a NanoDrop 2000c (Thermo Scientific Inc., Waltham, MA, USA). Total RNA (3 µg) of each sample was used to construct the cDNA library according to the Illumina instructions and then sequenced using an Illumina GA II (Illumina Inc., San Diego, USA). The original data processing and analysis methods were described in our previous study [72].

### 4.6. Quantitative Real-Time PCR Analyses

Leaf samples of the Arg7 variety subjected to MeJA, SA, ABA, NaCl, low temperature, mannitol, H_2_O_2_, or *Xam* treatments were collected to perform qRT-PCR analysis. Total RNA (1 µg) from each sample was used to synthesize the first-strand cDNA using oligo-dT primer by SuperScript reverse transcriptase (Takara, Dalian, China). The cDNA product was diluted to 50 ng·μL^−1^, and 1 μL was used for qRT-PCR. The qRT-PCR reaction mixtures (20 μL) contained 0.6 μL of each gene-specific primer (300 nmol·μL^−1^), 10 μL of 2× FastFire qPCR PreMix (Tiangen, Beijing, China), and 7.8 μL of RNase-free water. The qRT-qPCR thermal cycling included cDNA denaturation at 95 °C for 1 min, with 40 cycles of 95 °C for 5 s and 60 °C for 15 s in the Mx3005P Real-Time PCR System (Agilent Inc., Palo Alto, CA, USA) with the SYBR green method. The β-tubulin gene of cassava was chosen as an internal control. All qRT-qPCR experiments were performed in triplicate, and the gene-specific primers used in expression analysis are listed in Table S7. The data obtained from the qRT-qPCR were analyzed with Tukey’s post-hoc ANOVA in SPSS 22.0 (SPSS Inc., Chicago, USA) (*P* < 0.05) after fold treatment with the 2^−△△*C*t^ method.

## 5. Conclusions

In this study, we identified 91 PODs from the cassava genome and studied their basic classification, protein motif, gene structure, chromosomal distribution, and duplication pattern. Comprehensive transcriptional level analyses revealed the involvement of MePODs in biotic and abiotic stress responses, hormone responses, and storage root deterioration. Several *MePOD* genes (*MePOD-13, -17, -85,* and *-86*) were found to be transcriptionally upregulated after multiple different treatments, suggesting that these genes are good candidates to target for cassava improvement. These findings increase our understanding of POD-mediated stress and hormone responses and storage root deterioration in cassava, laying a foundation for the genetic improvement of cassava.

## Supplementary Materials

Supplementary materials can be found at https://www.mdpi.com/1422-0067/20/11/2730/s1. Table S1. Characteristics of PODs in cassava. Table S2. The Ka/Ks ratios of duplicated *POD* genes in cassava. Table S3. The expression profiles (log2-based values) of the cassava *POD* genes in different tissues. Table S4. The expression profiles (log2-based fold changes) of the cassava *POD* genes after drought treatment. Table S5. The expression profiles (log2-based fold changes) of the cassava *POD* genes after harvest. Table S6. The expression profiles (log2-based values) of the cassava *POD* genes after various stress treatments. Table S7. Primers used in qRT-PCR analysis. Figure S1. Phylogenetic analyses of 91 MePODs, 2 AtPRXs, 3 OsPRXs, and 3 ZmPRXs.
